# Supplementary material for: Assessing the durability and efficiency of landscape-based strategies to deploy plant resistance to pathogens
Source: PLoS Comput Biol. 2018 Apr 12;14(4):e1006067. doi: 10.1371/journal.pcbi.1006067 (PMC5918245; doi:10.1371/journal.pcbi.1006067)
Supplement: S1 Fig — (PDF) [file pcbi.1006067.s001.pdf]

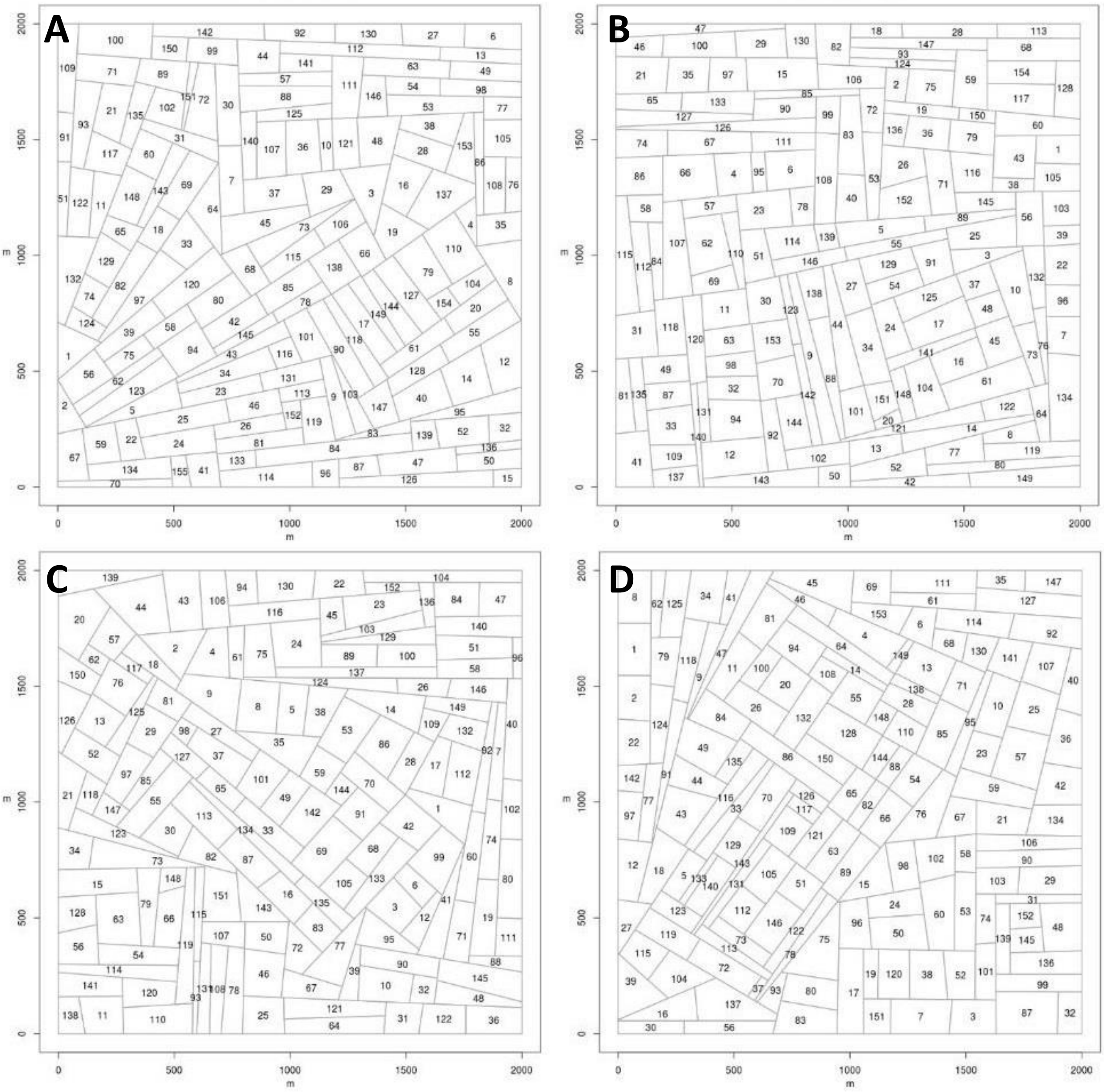

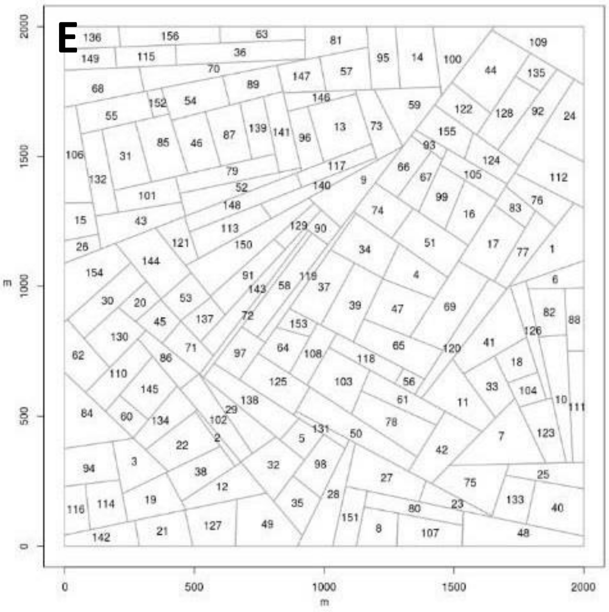

S1 Figure. Landscape structures used in this study. Landscapes of about 150 fields (total area: 2x2 km^2) were simulated using a T-tessellation algorithm from package RLiTe (http://kien-kieu.github.io/lite/rlite.html), with controlled number, area and shape of the fields. Numbers correspond to field indices. The effect of landscape structure was never statistically significant in our simulations.
