# Supplementary material for: Assessing the durability and efficiency of landscape-based strategies to deploy plant resistance to pathogens
Source: PLoS Comput Biol. 2018 Apr 12;14(4):e1006067. doi: 10.1371/journal.pcbi.1006067 (PMC5918245; doi:10.1371/journal.pcbi.1006067)
Supplement: S2 Fig — (PDF) [file pcbi.1006067.s002.pdf]

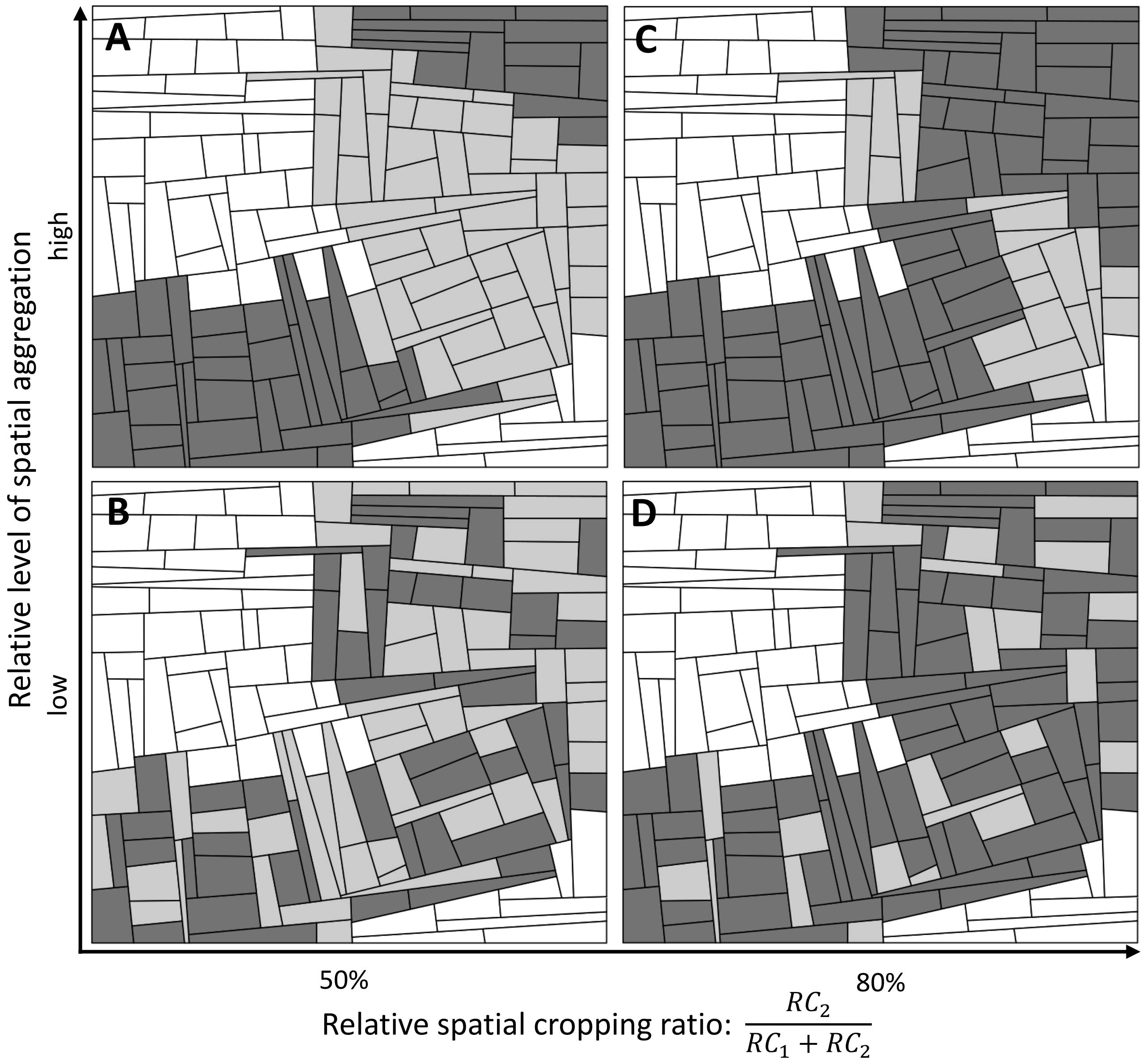

S2 Figure. Spatial allocation of three cultivars across a cropping landscape. A landscape structure is generated using T-tessellations. Then, a susceptible (white) and two resistant cultivars (RC_1, light grey, and RC_2, dark grey) are allocated to fields, with controlled relative proportions of the surface coverage (horizontal axis: 50% in A and B, 80% in C and D) and level of spatial aggregation (vertical axis: high in A and C, low in B and D) of RC_1 and RC_2. The total proportion of resistant fields is 2/3 and their level of aggregation is high.
