# Supplementary material for: Assessing the durability and efficiency of landscape-based strategies to deploy plant resistance to pathogens
Source: PLoS Comput Biol. 2018 Apr 12;14(4):e1006067. doi: 10.1371/journal.pcbi.1006067 (PMC5918245; doi:10.1371/journal.pcbi.1006067)
Supplement: S3 Fig — (PDF) [file pcbi.1006067.s003.pdf]

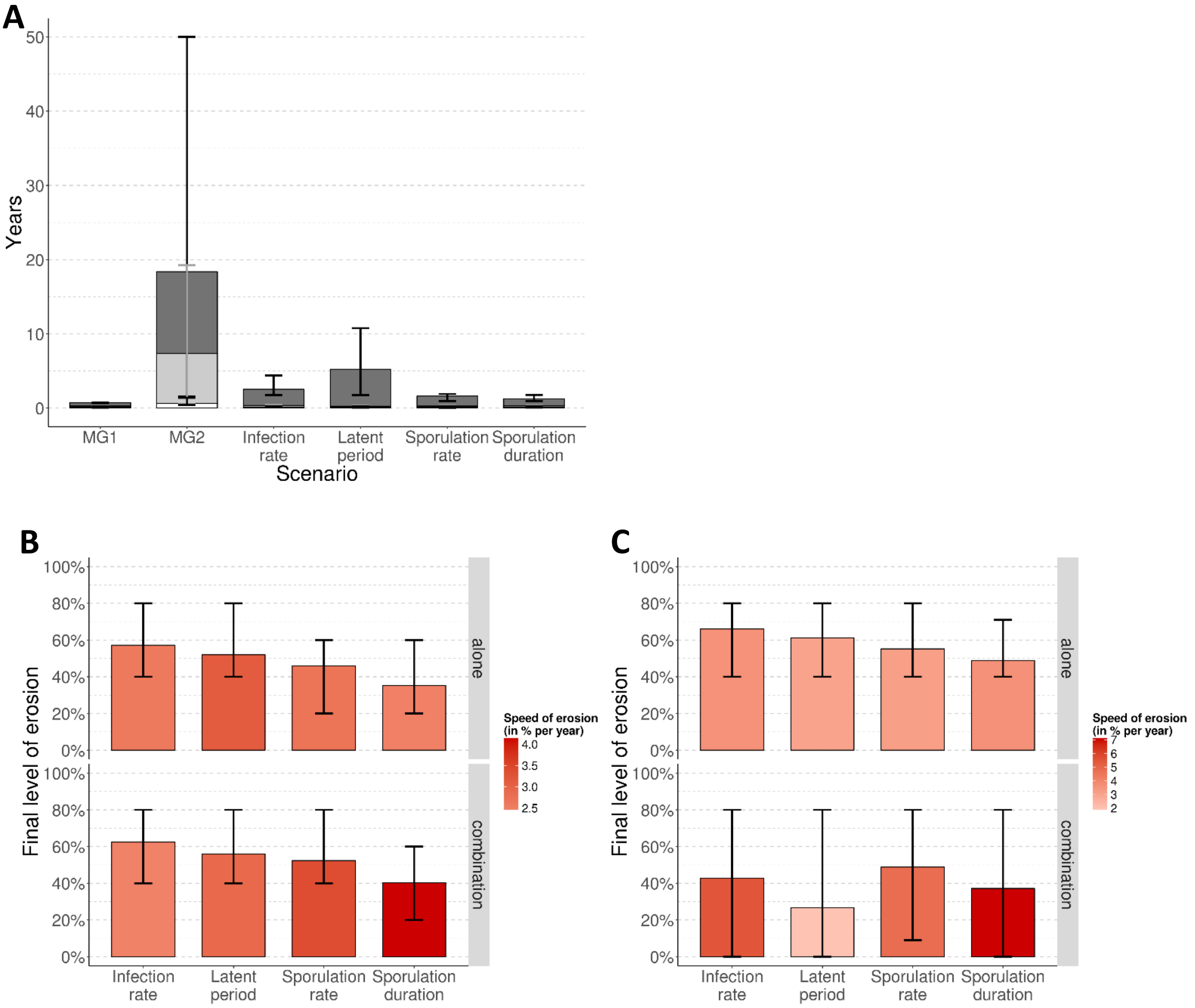

S3 Figure. Evolutionary outcome when quantitative resistance is highly effective. Here, the landscape is composed of a susceptible cultivar, and a resistant cultivar (cropping ratio: 80%) carrying a major gene (MG, efficiency 100%) or a quantitative resistant trait (efficiency 80% in A and B; 90% in C), alone or in combination. (A) Durability of the major gene: time to appearance of mutants (white), to first infection (light grey) or to establishment (dark grey) on resistant hosts. (B-C) Final level of erosion of quantitative resistance traits when deployed alone (top) or in combination with a major gene (bottom). The red shading indicates the average speed of erosion. Every scenario is replicated 50 times. Vertical lines show the 90% central range.
