# Supplementary material for: Assessing the durability and efficiency of landscape-based strategies to deploy plant resistance to pathogens
Source: PLoS Comput Biol. 2018 Apr 12;14(4):e1006067. doi: 10.1371/journal.pcbi.1006067 (PMC5918245; doi:10.1371/journal.pcbi.1006067)
Supplement: S4 Fig — (PDF) [file pcbi.1006067.s004.pdf]

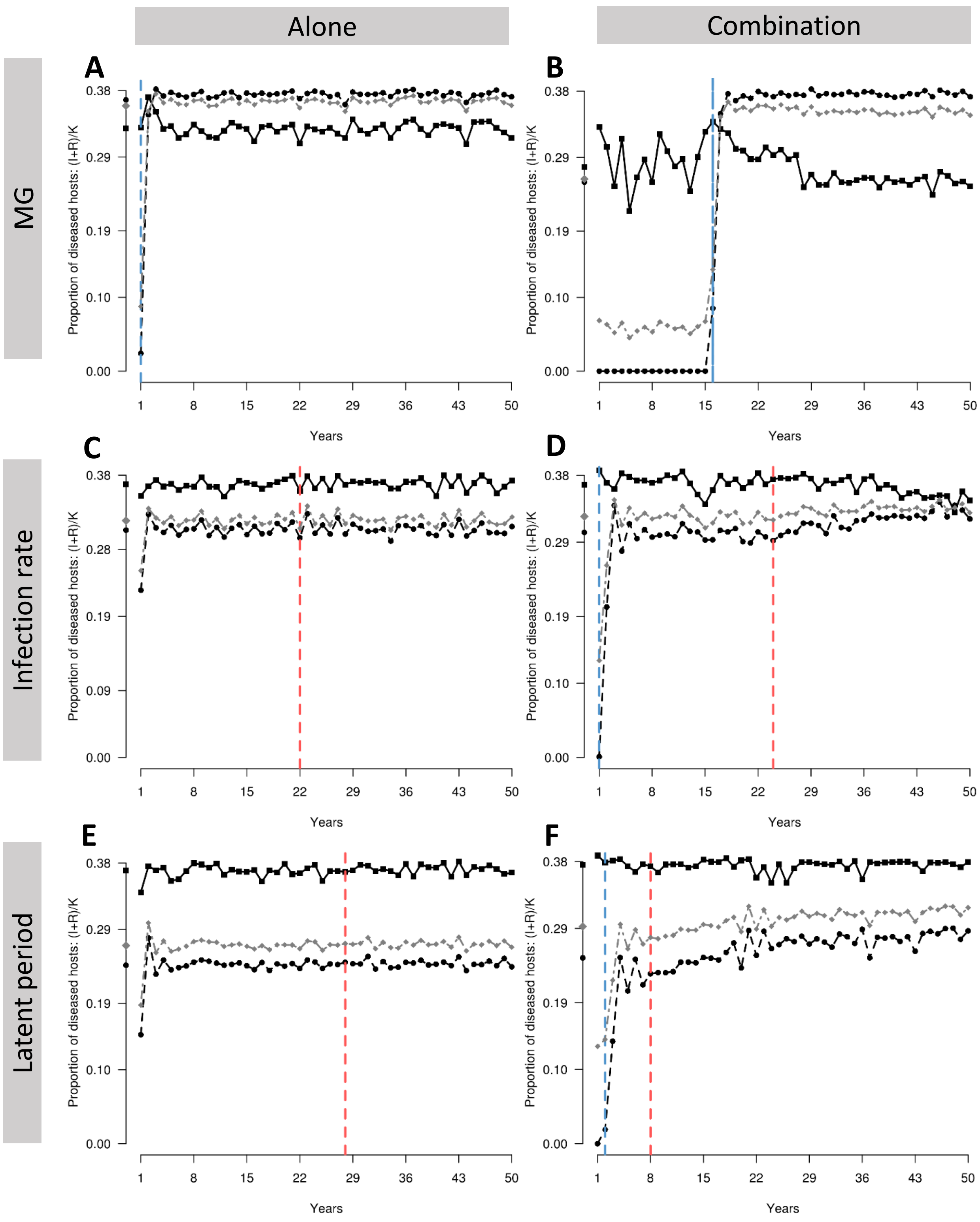

(see legend on next page)

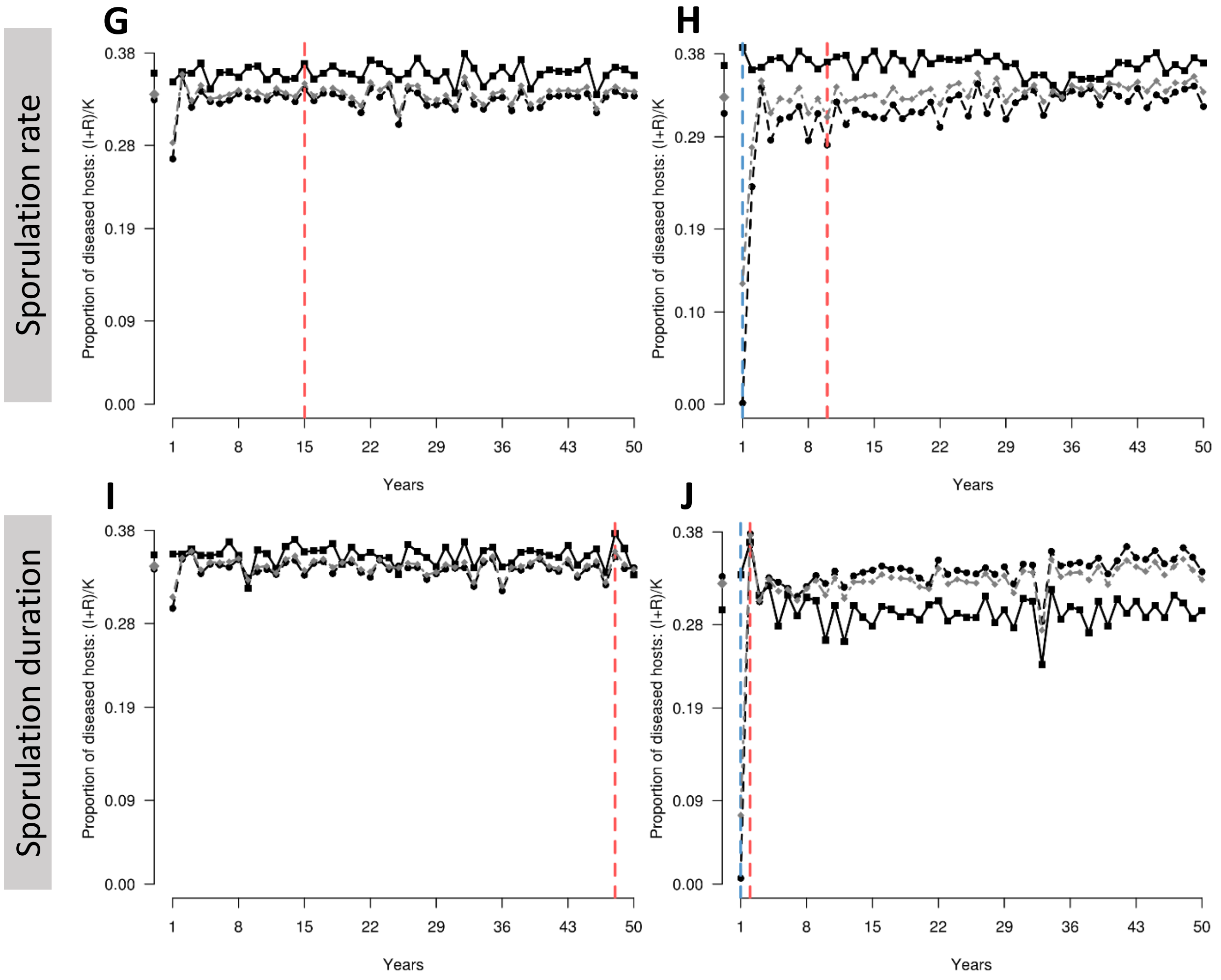

S4 Figure. Dynamics of diseased hosts from different scenarios of resistance deployment. Solid black curve: susceptible cultivar. Dashed black curve: resistant cultivar, carrying a major gene (A, B) or quantitative resistance against the infection rate (C, D), the latent period duration (E, F), the sporulation rate (G, H) or the sporulation duration (I, J), alone (A, C, E, G, I) or in combination with a major gene (B, D, F, H, J). Grey curve: whole landscape. The vertical blue lines mark the times to major gene breakdown; the vertical red lines mark the beginning of quantitative resistance erosion. The average AUDPC in a 100%-susceptible landscape is 0.38.
