# Supplementary material for: Assessing the durability and efficiency of landscape-based strategies to deploy plant resistance to pathogens
Source: PLoS Comput Biol. 2018 Apr 12;14(4):e1006067. doi: 10.1371/journal.pcbi.1006067 (PMC5918245; doi:10.1371/journal.pcbi.1006067)
Supplement: S6 Fig — (PDF) [file pcbi.1006067.s006.pdf]

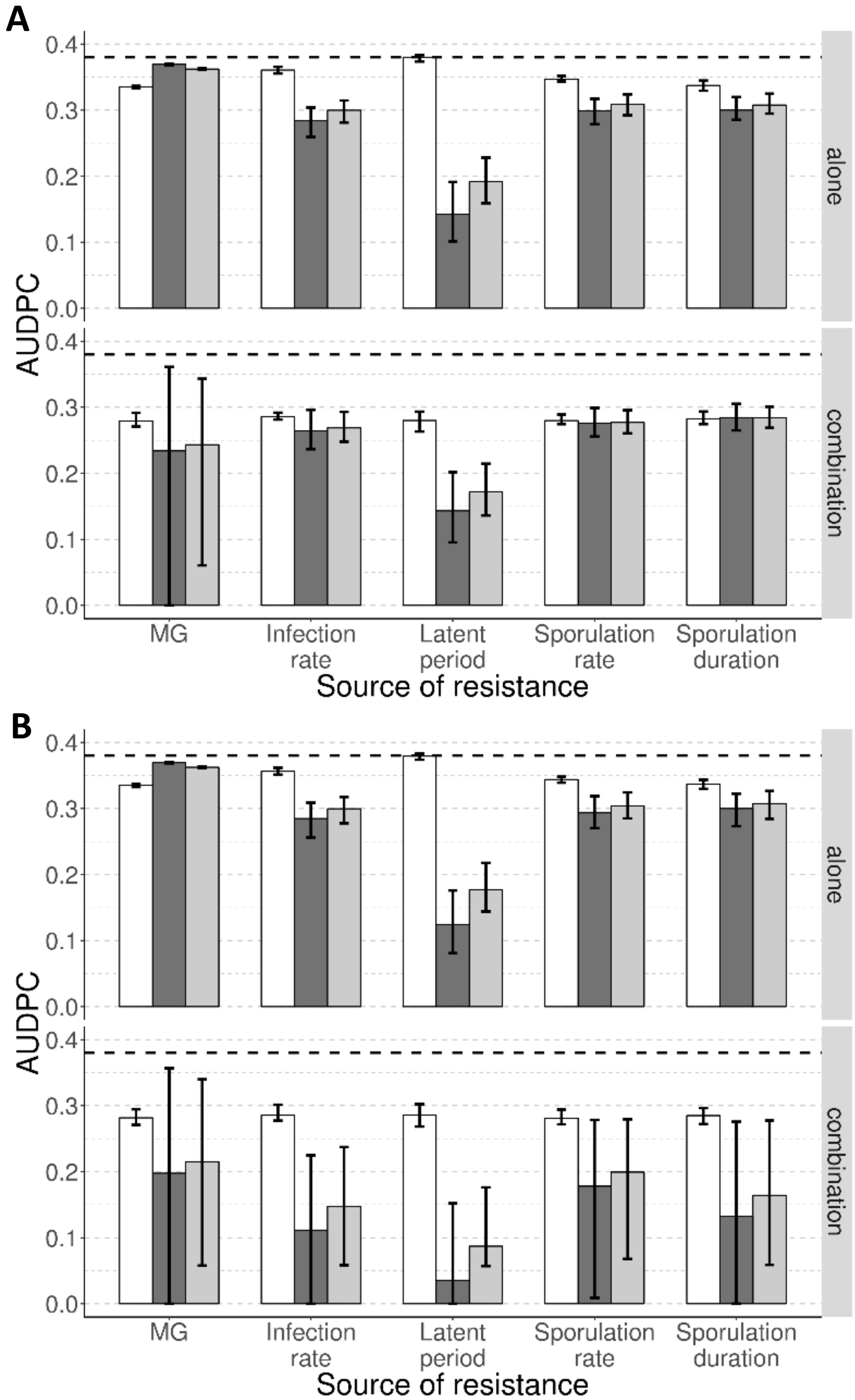

S6 Figure. Epidemiological outcome when quantitative resistance is highly effective. Here, the landscape is composed of a susceptible cultivar, and a resistant cultivar (cropping ratio: 80%) carrying a major gene (MG, efficiency 100%) or a quantitative resistant trait (efficiency 80% in A; 90% in B), alone (top) or in combination (bottom). Bars indicate the average area under disease progress curve (AUDPC) of the susceptible (white) and the resistant (dark grey) cultivars, as well as the whole landscape (light grey). The horizontal dashed line represents the average AUDPC in a fully susceptible landscape. Every scenario is replicated 50 times. Vertical lines show the 90% central range.
