# Supplementary material for: Assessing the durability and efficiency of landscape-based strategies to deploy plant resistance to pathogens
Source: PLoS Comput Biol. 2018 Apr 12;14(4):e1006067. doi: 10.1371/journal.pcbi.1006067 (PMC5918245; doi:10.1371/journal.pcbi.1006067)
Supplement: S1 Text — (PDF) [file pcbi.1006067.s007.pdf]

S1 Text. Model parameterisation to rust diseases

This section details the parameterisation of the model to approximate biotrophic foliar fungal diseases as typified by rusts of wheat, caused by fungi of the genus *Puccinia* (without targeting any specific rust species). Cited references are listed in the main text.

Pathogen aggressiveness components

Duration of latent and sporulation periods. We found 36 estimates from 29 studies of different rust pathogens which included information on the duration of the latent period; similarly, there were 18 estimates from 11 studies with data on the sporulation period. All of these estimates were derived from fully susceptible hosts grown in controlled conditions (S1 Table). Standardised conditions in growth chambers were preferred over field data to facilitate comparison between studies. When available, we selected estimates in which latent periods were measured as the time from inoculation until 50% of the total number of lesions began sporulating (the most robust measure of the latent period [75-77]), otherwise the time until appearance of the first sporulating lesion was used. A Gamma distribution was fit to the data using a maximum likelihood approach (S7 Figure). From this analysis, the parameters associated with latent and sporulation periods were estimated as: γ_{min}=10; γ_{var}=9; and Υ_{max}=24; Υ_{var}=105.

Infection rate. In an experiment on the dispersal of wheat brown rust, the probability that a propagule deposited on a leaf triggers an infection was estimated to lie between 0 and 0.4, depending on the propensity of the leaf to be infected [78]. In the present study, given that the propensity of a leaf to be infected is explicitly modelled by a sigmoid contamination function, the maximal expected infection rate was set at e_{max}=0.4.

Sporulation rate. The real number of spores produced daily by a lesion and effectively dispersed to a leaf where they may trigger an infection, often summarised by 'effective sporulation rate' and denoted as r_{max} here, is extremely complicated to estimate. Nevertheless, we found one study [79] where a field experiment on the spread of *P. striiformis* enabled estimation of $e_{max} \times r_{max}$ at 5 infections.day^{-1}. This would suggest that r_{max} should be 12.5 spores.day^{-1}. However, in our application case, this would represent an extremely aggressive pathogen. As an indication, the basic reproductive number for such a pathogen (i.e. the theoretical number of secondary infections from a single infectious host [80]) would be $R_{0\,max} = e_{max} \times \Upsilon_{max} \times r_{max} = 120$. Recently, the R_0 of *P. striiformis* in large fields has been estimated to be of the order of 30 [81]. Thus, in order to simulate a reasonably aggressive pathogen, and since very few data were available to quantify r_{max} compared to other parameters, r_{max} has been adjusted to be 3.125 spores.day^{-1}, thus $R_{0\,max} = 30$.

S1 Table. Available data on the duration of latent and sporulation periods for rust diseases caused by fungi of the genus *Puccinia*. In all these studies, both latent and sporulation periods were determined on fully susceptible hosts under controlled conditions. Different methods to estimate duration of the latent period were used by various authors: the time from inoculation until the appearance of the first sporulating lesion (red cells), or until 50% of the lesions began sporulating (green cells). When the method is unspecified, the background of the cell is left blank.

Reference	*Organism*	Disease	Latent period (days)			Sporulation period (days)		
			min	max	mean	min	max	mean
[82]	*P. coronata*	Oat crown rust	6.00	10.00	8.00			
[83]	*P. coronata*	Oat crown rust	6.00	10.00	8.00	11.0	16.0	13.5
[83]	*P. graminis*	Oat stem rust	6.00	10.00	8.00	17.0	24.0	20.5
[84]	*P. graminis*	Wheat stem rust	12.25	7.25	9.75			
[85]	*P. graminis*	Wheat stem rust	8.00	14.00	11.00			
[83]	*P. graminis*	Wheat stem rust	6.00	10.00	8.00	17.0	24.0	20.5
[86]	*P. hordei*	Barley leaf rust			5.00			
[83]	*P. hordei*	Barley leaf rust	6.00	10.00	8.00	17.0	24.0	20.5
[87]	*P. hordei*	Barley leaf rust	5.30	13.50	9.40	14.1	29.5	21.8
[83]	*P. recondita*	Triticale brown rust	6.00	10.00	8.00	11.0	16.0	13.5
[88]	*P. recondita*	Wheat brown rust	6.00	10.00	8.00	52.0	64.0	58.0
[89]	*P. recondita*	Wheat brown rust			8.00			21.0
[83]	*P. recondita*	Wheat brown rust	6.00	10.00	8.00	17.0	24.0	20.5
[90]	*P. recondita*	Wheat brown rust			8.00	20.0	27.0	23.5
[91]	*P. recondita*	Wheat brown rust	8.30	10.10	9.20	17.8	33.9	25.9
[92]	*P. striiformis*	Barley stripe rust	12.00	14.00	13.00			
[93]	*P. striiformis*	Barley stripe rust	9.00	15.70	12.35			
[94]	*P. striiformis*	Wheat stripe rust	12.20	13.95	13.08			
[95]	*P. striiformis*	Wheat stripe rust	12.60	13.10	12.85			
[96]	*P. striiformis*	Wheat stripe rust	12.03	14.27	13.15			
[97]	*P. striiformis*	Wheat stripe rust	13.00	15.10	14.05			
[98]	*P. striiformis*	Wheat stripe rust	10.00	24.00	17.00	9.0	13.0	11.0
[99]	*P. striiformis*	Wheat stripe rust	6.10	13.00	9.55			
[100]	*P. striiformis*	Wheat stripe rust	11.40	15.10	13.25			
[101]	*P. striiformis*	Wheat stripe rust			11.20			
[102]	*P. striiformis*	Wheat stripe rust	12.40	19.20	15.80			
[89]	*P. striiformis*	Wheat stripe rust			10.00		>29	29.0
[83]	*P. striiformis*	Wheat stripe rust	6.00	10.00	8.00	17.0	24.0	20.5
[79]	*P. striiformis*	Wheat stripe rust			17.00			14.0
[103]	*P. striiformis*	Wheat stripe rust	14.80	19.20	17.00			
[104]	*P. triticina*	Wheat leaf rust	8.20	9.30	8.75			
[105]	*P. triticina*	Wheat leaf rust	7.00	10.00	8.50			
[106]	*P. triticina*	Wheat leaf rust			7.75			
[107]	*P. triticina*	Wheat leaf rust	6.00	8.00	7.00	20.0	30.0	25.0
[108]	*P. triticina*	Wheat leaf rust			9.00			30.0

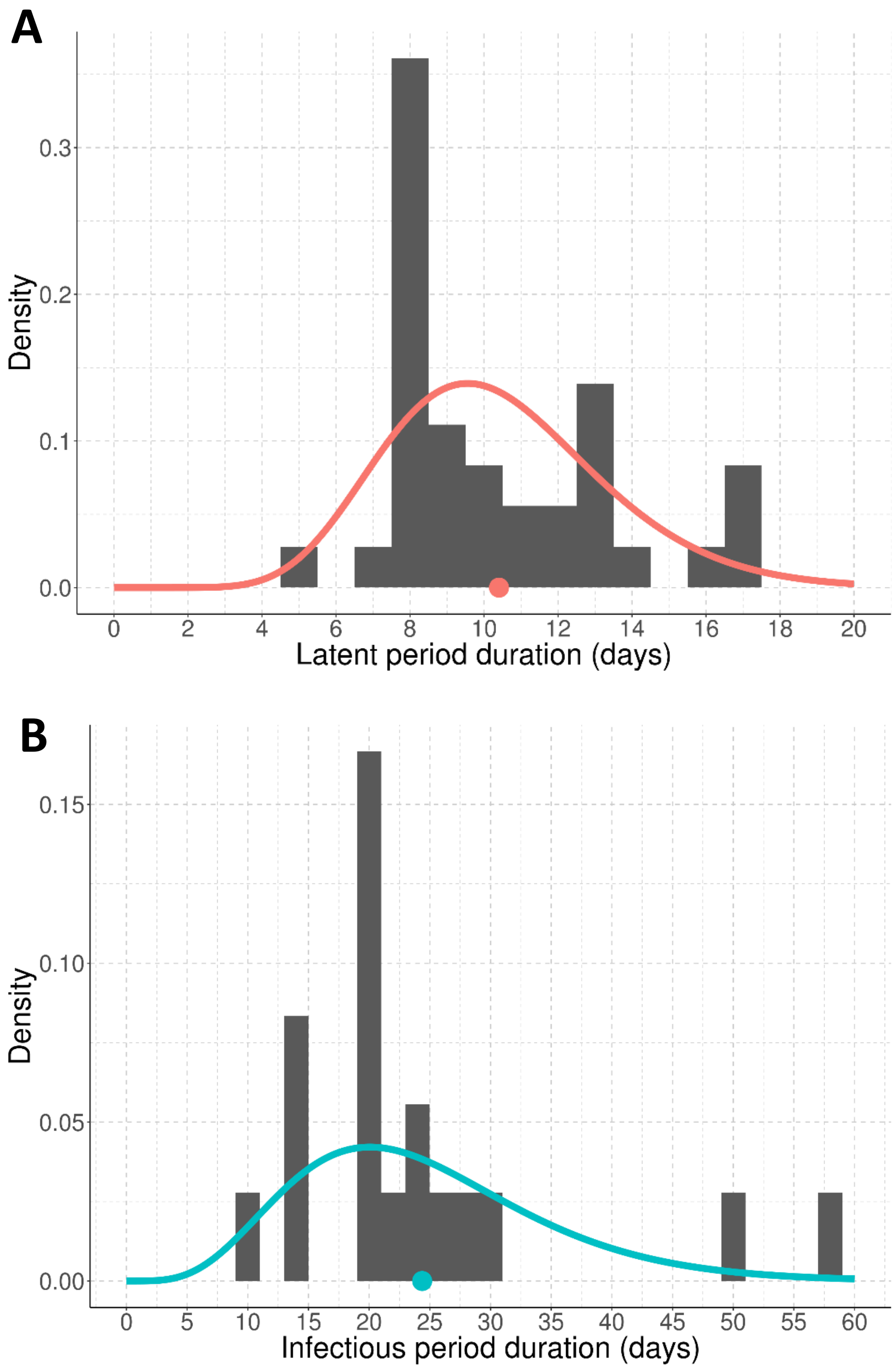

S7 Figure. Distribution of the latent period duration (A) and length of the sporulation period (B) of rust diseases caused by fungi of the genus *Puccinia*. Raw data were obtained from studies estimating these parameters on fully susceptible cereal cultivars (see S1 Table). Red and blue curves are Gamma distributions estimated from the data through maximum of likelihood (function *fitdistr* of the R package MASS, v7.3-47); dots indicate the mean latent period (red, 10.41 days) and sporulation period (blue, 24.37 days). The variance is 8.80 days for the latent period and 105.12 days for the sporulation period.

Pathogen dispersal

Based on the results of previous studies designed to estimate the dispersal kernels of *P. lagenophorae* [109] and *P. striiformis* [81, 110], the power-law function has a good ability to predict the dispersal of rust spores, assuming it is isotropic (i.e. uniform in all directions). We consequently use this function in our model:

$$g(\|z' - z\|) = \frac{(b-2)(b-1)}{2.\pi.a^2}.\left(1 + \frac{\|z'-z\|}{a}\right)^{-b} \quad (23)$$

where a > 0 is a scale parameter and b > 2 determines the weight of the dispersal tail. The expected dispersal distance is given by: $\mu_{exp} = \frac{2a}{(b-3)}$.

However, the estimation of parameters a and b is not straightforward. Indeed, many studies dealing with the dispersal of rust spores did not use a power-law function [78, 111], or they did but used a unidimensional or modified power-law functions [81, 109, 110, 112, 113], which makes calibration of our own function difficult. We therefore focused on the results obtained in four studies [78, 109, 110, 113] which used exponential functions as dispersal kernels. In spite of a lower goodness of fit of the exponential function to model spore dispersal [81, 110, 113], estimation of a mean dispersal distance from this function is easier and enables direct comparison between studies. The overall mean dispersal distance in these four studies is 19.46 m (S2 Table), thus we calibrated a and b of our power-law function in such a way that μ_{exp}=20 m, which represents 1% of landscape length. Among the possible solutions we arbitrarily chose a=40 and b=7 (S8 Figure).

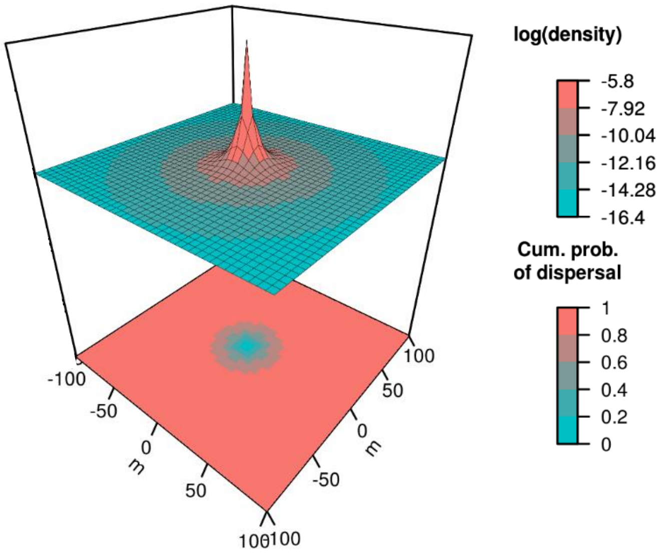

S8 Figure. Two-dimensional representation of the power-law dispersal kernel used in this study (μ_{exp}=20 m; a=40; b=7.). The top panel indicates the logarithm of the probability to disperse from the origin to any point of the landscape, and the bottom panel indicates the cumulative probability to disperse over a given distance.

S2 Table. Mean dispersal distance of rust spores computed from available results obtained in studies which used exponential functions. Let the dispersal function be written as $f(x) = b.e^{-\frac{x}{a}}$ with x the distance of dispersal. Then the mean dispersal distance is simply $\mu_0 = 2.a$.

Reference	Pathogen	Mean dispersal distance (m)
[113]	*P. graminis*	36.50
	P. graminis	8.51
	P. graminis	94.76
	P. polysora	18.18
	P. polysora	11.11
	P. sorghi	2.22
[109]	*P. lagenophorae*	0.40
[110]	*P. striiformis*	18.87
	P. striiformis	1.99
	P. striiformis	13.42
	P. striiformis	8.16
	P. striiformis	3.04
	P. striiformis	1.91
	P. striiformis	1.98
	P. striiformis	0.66
[78]	*P. recondita*	38.60
	P. recondita	34.00
	P. recondita	27.80
	P. recondita	38.00
	P. recondita	29.00
	**Overall mean**	**19.46 m**

The parameters of the sigmoid curve for contamination of hosts by propagules (κ=5.33, σ=3) have been parameterised as in previous works [40, 42, 55], such as π(0)=0, π(1)=1, and the inflexion point is located at $x_0 = ((\sigma - 1)/\kappa\sigma)^{1/\sigma} \approx 0.5$. Thus, the contamination of a healthy host is easier when the proportion of healthy hosts is higher than 50%, and harder otherwise (S9 Figure).

Initial conditions and seasonality

The host dynamics (host growth, δ_v = 0.1 day^{-1}; plantation density, C_v^0 = 0.1 m^{-2}; maximal density, C_v^{max} = 2 m^{-2}) was parameterised as in previous works [45], in order to obtain reasonable host dynamics in the absence of disease (GLA_{TOT}=1.48 day^{-1}.m^{-2}, noting that host dynamics is deterministic, S10 Figure).

The probability of infection at the beginning of the simulation (ϕ=5.10^{-4}) and the off-season survival probability (λ=10^{-4}) have been set such that epidemics in a fully susceptible landscape are regular (average of 10 simulations: GLA_{TOT}=0.48 day^{-1}.m^{-2} [standard deviation: 0.005], $AUDPC_{TOT}$=0.38 [0.001], see the output of one simulation in S11 Figure).

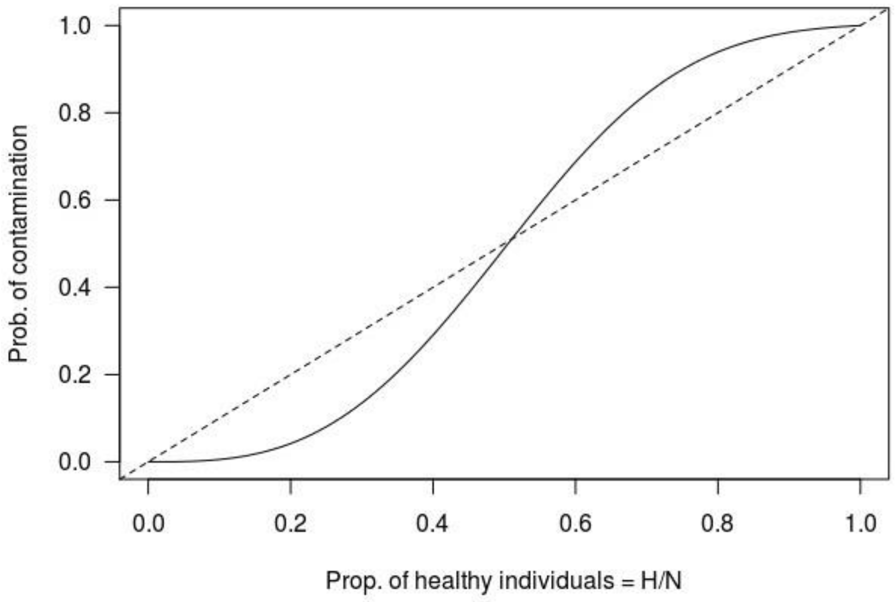

S9 Figure. Sigmoid contamination function. Probability for a healthy host to be contaminated following propagule arrival. The equation is $\pi(x) = \frac{1-e^{-\kappa . x^{\sigma}}}{1-e^{-\kappa}}$ with κ=5.33 and σ=3.

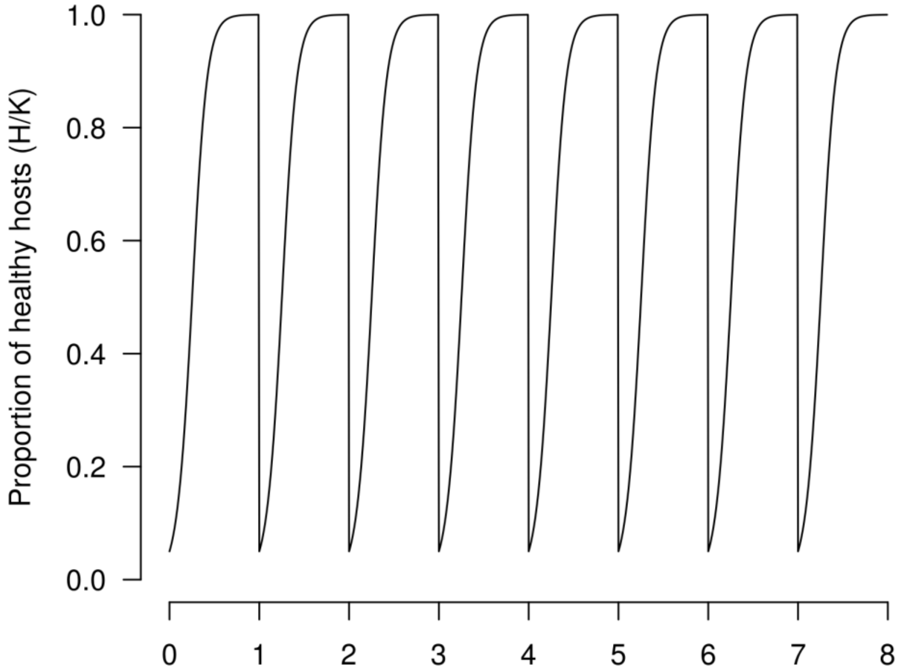

S10 Figure. Deterministic dynamics of healthy hosts in the absence of disease. GLA_{TOT}=1.48 $day^{-1}.m^{-2}$.

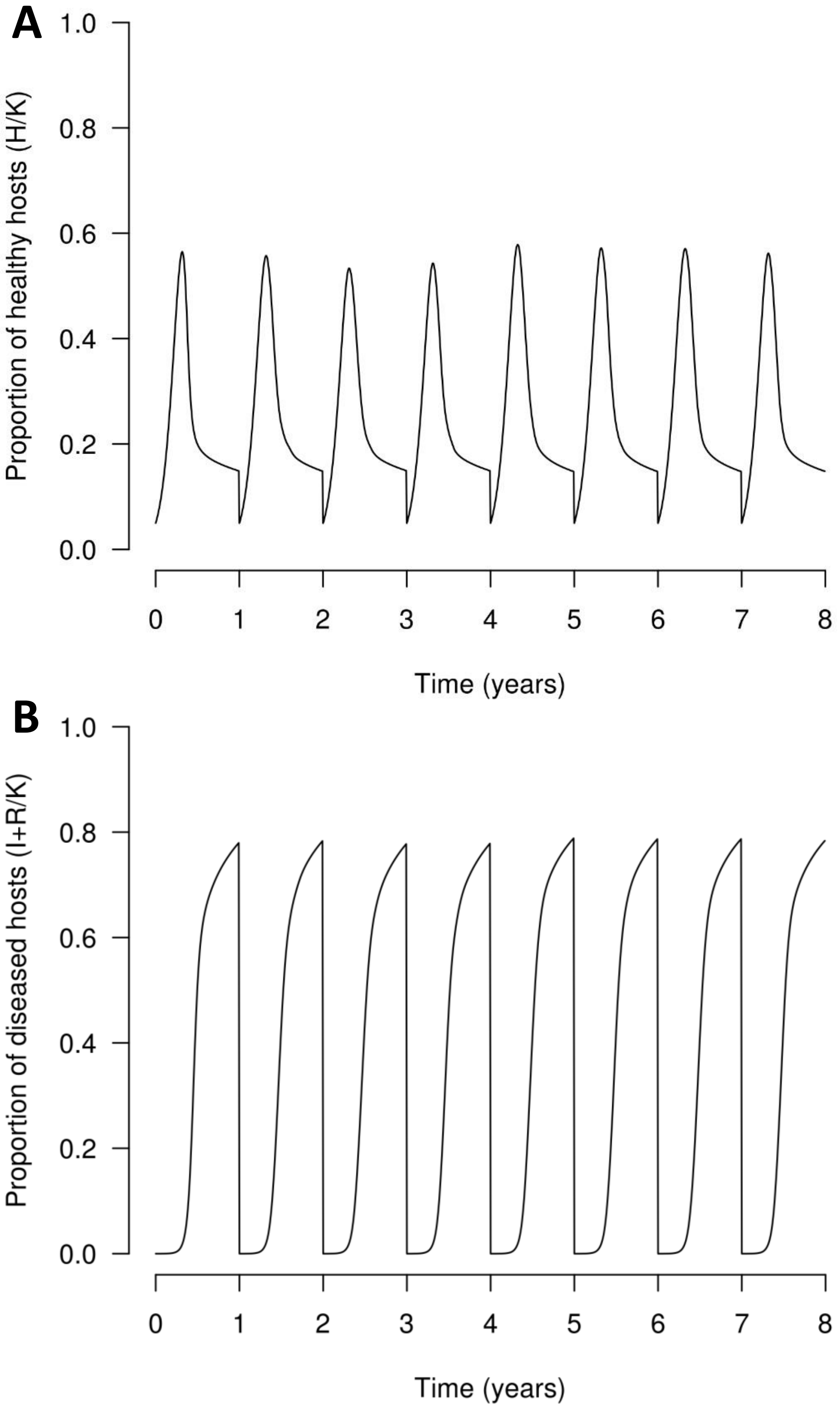

S11 Figure. Dynamics of healthy (A, GLA_{TOT}=0.49 day^{-1}.m^{-2}) **and diseased** (B, $AUDPC_{TOT}$=0.38) **hosts in a fully susceptible landscape in a simulated example.**

Evolutionary parameters

Very few empirical data are available from rust pathogens to help parameterise the model with respect to mutation probabilities, cost of infectivity, cost of aggressiveness and number of mutations required to completely erode a trait for quantitative resistance. It is important to remember that the mutation probability in the model refers to the probability that a spore has a different phenotype from its parental lesion. For instance, τ_1 gives the probability for a spore to have an infective phenotype on a resistant cultivar carrying major gene 1, given that this spore was produced by a lesion triggered by a non-infective pathogen. This probability depends on the number of mutations per generation per base pair (i.e. the classic 'genetic mutation rate' of empirical studies), the number and nature of the specific genetic mutations required to overcome major gene 1, and the potential dependency between these mutations. In addition to the lack of empirical data, all these parameters are likely to vary depending on the pathosystem as well as the resistance sources.

Consequently, this work focuses on a simple theoretical case, where evolutionary parameters have been arbitrarily fixed. Since one of the objectives of this application case is to identify promising combinations of qualitative and quantitative resistance, and based on preliminary simulations, the mutation probabilities were set at $\tau_g=\tau_w=10^{-4}$ in such way that a cultivar carrying a single major gene would be overcome in less than one year. Using these values, pathogen evolution is quick enough to potentially adapt to the resistance genes, but slow enough to allow comparison of the effectiveness of different resistance combinations with regard to controlling disease. The resistance efficiency is set at $\rho_g=1$ for qualitative resistance (i.e. a major resistance gene confers complete immunity against non-adapted pathogens), and $\rho_w=0.5$ for quantitative resistance traits (i.e. infection rate, sporulation rate, duration of the sporulation period, or number of epidemic cycles in a cropping season of a non-adapted pathogen on resistant hosts is reduced by 50%). The cost of infectivity and the cost of aggressiveness were set at $\theta_g=\theta_w=0.5$, the trade-off strength at $\beta_w=1$ (linear trade-off), and the number of pathotypes with regard to the aggressiveness components at $Q_w=6$ (i.e. every mutation step improves or degrades an aggressiveness component by 20%). This last value is a compromise between the high number of steps required to simulate a gradual pathogen evolution, and having a smaller number of different pathotypes to limit the computational time required to perform the simulations.

This parameterisation gives the following infectivity and aggressiveness matrices:

**Infectivity matrix for major gene g** INF^g		**Host genotype v**	
		Susceptible (SC) $mg_g(v)=0$	Resistant (RC) $mg_g(v)=1$
**Pathogen genotype p**	Non-infective $ig_g(p)=0$	1	0
**Pathogen genotype p**	Infective $ig_g(p)=1$	0.5	1

**Aggressiveness matrix for component w** AGG^w		**Host genotype v**	
		Susceptible (SC) $qr_w(v)=0$	Resistant (RC) $qr_w(v)=1$
**Pathogen genotype p**	Non-aggressive $ag_w(p)=1$	1	0.5
**Pathogen genotype p**	$ag_w(p)=2$	0.9	0.6
**Pathogen genotype p**	$ag_w(p)=3$	0.8	0.7
**Pathogen genotype p**	$ag_w(p)=4$	0.7	0.8
**Pathogen genotype p**	$ag_w(p)=5$	0.6	0.9
**Pathogen genotype p**	Fully aggressive $ag_w(p)=6$	0.5	1

Grey lines indicate pathogen population at the beginning of the simulations.

The parameterisation of the mutation probabilities ($\tau_g=10^{-4}$ and $\tau_w=10^{-4}$) and the number of steps to completely erode a quantitative resistance (given by Q_w) give the following mutation matrix for infectivity gene g:

	$ig_g(p)=0$	$ig_g(p)=1$
$ig_g(p)=0$	0.9999	10^{-4}
$ig_g(p)=1$	10^{-4}	0.9999

and the following mutation matrix for aggressiveness component w:

	$ag_w(p)=1$	$ag_w(p)=2$	$ag_w(p)=3$	$ag_w(p)=4$	$ag_w(p)=5$	$ag_w(p)=6$
$ag_w(p)=1$	0.9999	10^{-4}	0	0	0	0
$ag_w(p)=2$	5.10^{-5}	0.9999	5.10^{-5}	0	0	0
$ag_w(p)=3$	0	5.10^{-5}	0.9999	5.10^{-5}	0	0
$ag_w(p)=4$	0	0	5.10^{-5}	0.9999	5.10^{-5}	0
$ag_w(p)=5$	0	0	0	5.10^{-5}	0.9999	5.10^{-5}
$ag_w(p)=6$	0	0	0	0	10^{-4}	0.9999

Discussion of model parameterisation and assumptions

Parameterisation to rust pathogens.

Pathogen aggressiveness. This model has been calibrated for rust-like pathogens using available knowledge of the epidemiology of fungi in the genus *Puccinia*. In particular, we found many data related to pathogen aggressiveness and dispersal. This allows the reliable estimates of parameters e_{max}, γ_{min}, γ_{var}, Υ_{max} and Υ_{var}. However, as noted above, the calculation of the effective number of spores produced daily by a lesion and effectively dispersed to a leaf where they may trigger an infection (r_{max} in our model) is extremely challenging. Acquisition of data to inform this parameter is crucial, since this parameter has a strong influence on epidemic spread and pathogen evolution [45]. In this study, r_{max} directly affects pathogen population size, which influences both the number of new infected hosts from a single infectious source, and the probability that some of these new infections are triggered by mutant spores. For example, smaller values should lead to higher times to appearance of mutants (durability measure (d1)) than what we observed in our simulations (see white bars in Fig 5A).

Pathogen dispersal. The dispersal of fungal spores has been extensively studied, and we now have considerable evidence to support the use of a power-law kernel to simulate spore dispersal [81, 109, 110]. Available data indicate that the mean dispersal distance is of the order of 20 m (see S2 Table). However, sensitivity analyses of models simulating plant epidemics show that both the mean dispersal distance [40, 114] and the width of the tail of the dispersal kernel [42, 79] have a great impact on pathogen spread. In the present work, the calibration of parameters a and b gives an appropriate mean dispersal distance, but is probably less accurate with respect to simulation of long-distance dispersal events (determined by the width of the tail of the dispersal kernel). In the future, we need studies built with exactly the same equations, to better compare and calibrate parameter values of dispersal kernels. This will be particularly important in future modelling studies aiming to explore the effect of landscape composition (e.g. proportion and spatial aggregation of a resistant cultivar) on pathogen spread and evolution (see 'Future research directions' in the discussion section).

All healthy sites of a plant do not have the same propensity to be infected [78]. This is at least partly due to plant architecture, which makes healthy sites not equally accessible to spores following dispersal. Thus, once the most accessible sites are infected, the remaining healthy sites may be harder to reach and infect. Our contamination function (see S9 Figure) accounts for this process. The high flexibility of this function makes it relevant for a wide range of pathogens, including rusts. However, we did not have any experimental data to parameterise it for cereal crops. Therefore, we used the same arbitrary values (κ, σ) as in previous works [40, 55] in order to facilitate comparisons between studies. Nevertheless, since this function only mitigates the overall infection rate (e_{max}), it is likely that epidemic spread depends more on the value of the latter than on the parameters or shape of this sigmoid function. A sensitivity analysis of a similar simulation model showed that use of a linear contamination function, rather than a sigmoid function [41] has little effect on the level of pathogen adaptation to quantitative resistance.

Seasonality and initial conditions. Parameters related to host growth (δ_v), host density (C_v^{max}), initial conditions (C_v^0 and ϕ), and seasonality (λ) are specific to the agricultural (e.g. choice of cultivars, planting density) and epidemiological (e.g. initial level of contamination, presence of a wild reservoir or volunteer host plants for the pathogen) contexts. In our application case, these parameters have

been parameterised so as to simulate regular host dynamic and epidemics. This helps compare different resistance deployment strategies in standardised conditions, and attribute variation in model outputs to the deployment strategies rather than to the initial conditions or the epidemiological context. On the other hand, it could be interesting to vary the growth rate of resistant cultivars (or their contribution to yield) to study the impact of cost of resistance on the performance of different deployment strategies. One could also vary the off-season survival probability of infectious hosts (λ) to investigate the potential of strategies based on green bridge suppression (e.g. fungicide treatments in the end of the cropping season; removal of potential volunteer plants during the off-season).

Pathogen evolution. As discussed previously, parameterisation of evolutionary processes in the model from empirical data is extremely difficult. In addition to the scarcity of studies designed to estimate such parameters for rusts, these parameters may not correspond with the definition of the parameters in our model (e.g. our 'mutation probability' is different from the classic 'mutation rate'; see above). Consequently, we chose to investigate combinations of qualitative and quantitative resistances in a simple context, using the same mutation probabilities (τ_g and τ_w), costs of infectivity (θ_g) and aggressiveness (θ_w), trade-off strengths (β_w), and numbers of steps to completely erode a quantitative resistant trait (Q_w) for every major resistance gene g and trait of quantitative resistance w.

In this model, the mutation probability gives the probability of appearance of mutants. Therefore it has a great effect on the time to appearance of such mutants [30, 39] or the speed of erosion of quantitative resistance [56]. However, provided that mutation probabilities are the same for all infectivity genes and aggressiveness components (i.e. $\tau_g = \tau_w = constant, \forall g, \forall w$), the specific value of these mutation probabilities should similarly affect all scenarios of our numerical experimental design. In other words, they should not change the relative durability and epidemiological efficiency of these simulated scenarios, and consequently the ranking of the tested resistance combinations. To test this hypothesis, we replicated the simulations with smaller mutation probabilities: $\tau_g = \tau_w = \{10^{-5}; 10^{-6}; 10^{-7}\}$. The results of these new simulations show that the durability of the major gene increases with decreasing mutation probabilities (S5ACE Figure), but the tested resistance combinations keep the same ranking. Similarly, the combinations of qualitative and quantitative resistances result in better epidemiological outcomes when the mutation probabilities decrease (S5BDF Figure). Nevertheless, regardless the mutation probabilities, the most promising combination is still the pyramid of two major resistance genes, followed by a major gene combined with a quantitative resistance trait against the latent period, next with a quantitative resistance trait against the infection rate and the sporulation rate, and finally with a quantitative resistance trait against the sporulation duration.

In contrast, the fitness costs associated with pathogen adaptation may differentially affect the simulated scenarios. Several studies have demonstrated that higher infectivity costs increase resistance durability [29, 30] and epidemiological efficiency [33]. In our results, the durability of the pyramid of two major genes is likely due to the cost of infectivity (θ_g) endured by mutant pathogens (see main text and Fig 5A). However, smaller values would result in a reduced performance of this strategy in comparison to other resistance combinations. This may also be true for comparisons of pyramiding with other spatio-temporal deployment strategies such as mosaics [50]. Similarly, the time to establishment of mutant pathogens in a host population carrying quantitative resistance has been shown to increase with higher costs of aggressiveness [37] or stronger trade-off relationships [41, 45,

55]. Thus, the associated parameters (θ_w and β_w), as well as the number of steps to erode a quantitative resistant trait (Q_w) should considerably impact model outputs like final level and speed of erosion of quantitative resistance.

To conclude, the impact of all these evolutionary parameters, related to the choice of the resistance source, on the evolutionary and epidemiological outcomes of the simulated deployment strategies need to be explicitly evaluated within a dedicated numerical experimental design. This is the objective of future studies (see section 'Future research directions' in main text). Furthermore, given the variability in fitness costs and trade-off relationships associated with the evolution of pathogens, including rust fungi [65], a careful assessment of these parameters is required before the deployment of genetic resistance in the field, in order to get reliable predictions of their durability and efficiency.

Parameterisation to necrotrophic fungal pathogens.

Parameterisation of our model is flexible enough to encompass a wide range of pathogens. As an example, although our application case focused on biotrophic fungi, necrotrophic pathogens could be simulated, by allowing transmission from stubble after crop harvest instead of from living tissue during the cropping season. To this end, the latent period could be increased to cover the whole cropping season, and the off-season survival probability could be adjusted in such a way that the total pool of produced spores is available for the following cropping season (after which the pool of spores would no longer be available). This scenario would at least partially represent major features of interactions such as that between canola and blackleg (*Leptosphaeria maculans*) [115].
