# Supplementary material for: Assessing the durability and efficiency of landscape-based strategies to deploy plant resistance to pathogens
Source: PLoS Comput Biol. 2018 Apr 12;14(4):e1006067. doi: 10.1371/journal.pcbi.1006067 (PMC5918245; doi:10.1371/journal.pcbi.1006067)
Supplement: S2 Text — (PDF) [file pcbi.1006067.s008.pdf]

# S2 Text. Calculation of the threshold for pathogen establishment.

In this work, the time to mutant pathogen establishment in the resistant host population is defined as the point at which extinction becomes unlikely. Assessment of this time requires the calculation of a threshold (denoted by N hereafter) for the number of infections of resistant hosts by mutant pathogens, above which mutant pathogens are unlikely to go extinct.

The spread of a mutant pathogen across years from a single infection of a resistant host requires surviving the bottleneck imposed by host harvest (denoted by event $\{Surv_I\}$), and the infection of new resistant hosts at the beginning of the next cropping season (denoted by event $\{Inf_I|Surv_I\}$). Thus the probability of effective spread from this single infection (event $\{Inf_I\}$) is:

$$P(Inf_I) = P(Surv_I) \times P(Inf_I|Surv_I) \quad (1)$$

The probability for a single infection to survive the bottleneck imposed by host harvest and the off-season is simply:

$$P(Surv_I) = \lambda \quad (2)$$

We now assume that a single infection survives the bottleneck. The probability for a single spore, produced by a mutant pathogen (completely adapted to the resistant host population) to infect a resistant host (assumed to be present in the field) is given by $e_{max}$ (neglecting the probability of reverse mutation to a non-adapted pathotype, the probability to disperse outside the field, and the effect of plant architecture on the probability of contamination). Then, the probability of extinction of this single spore is $1 - e_{max}$.

Since a single infection produces a total of $r_{max} \times \Upsilon_{max}$ spores, the probability of extinction of all these spores is: $P(Ext_{Pr}) = (1 - e_{max})^{r_{max} \times \Upsilon_{max}}$. Given the values of $e_{max}$=0.40, $r_{max}$=3.125 and $\Upsilon_{max}$=24, we have $P(Ext_{Pr}) \approx 2.3 \times 10^{-17}$. Consequently, the probability of infection of new resistant hosts by a single infection is:

$$P(Inf_I|Surv_I) = 1 - P(Ext_{Pr}) \approx 1 \quad (3)$$

The combination of equations (1), (2) and (3) gives:

$$P(Inf_I) = \lambda \quad (4)$$

Then, the probability of extinction of a single infection is $P(Ext_I) = 1 - P(Inf_I) = 1 - \lambda$, and the probability of extinction of N infections is:

$$P(Ext) = P(Ext_I)^N = (1 - \lambda)^N \quad (5)$$

S12 Figure shows the probability of extinction for different values of N, with $\lambda=10^{-4}$. Above N=50,000 infections, the probability of extinction is less than 1%. Therefore, we chose this threshold to define the time to mutant pathogen establishment.

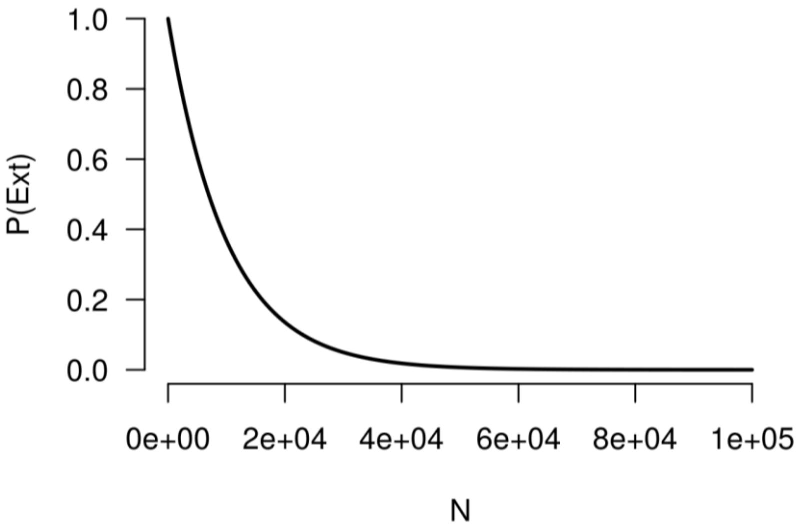


**S12 Figure. Probability of extinction of a mutant pathogen in a steady environment, depending on the number of infections.** $P(Ext) = (1 - \lambda)^N$ with $\lambda = 10^{-4}$
